# Dietary restriction protects against diethylnitrosamine-induced hepatocellular tumorigenesis by restoring the disturbed gene expression profile

**DOI:** 10.1038/srep43745

**Published:** 2017-03-06

**Authors:** Ting Duan, Wenjie Sun, Mohan Zhang, Juan Ge, Yansu He, Jun Zhang, Yifan Zheng, Wei Yang, Han-ming Shen, Jun Yang, Xinqiang Zhu, Peilin Yu

**Affiliations:** 1 Department of Toxicology, School of Public Health, Zhejiang University, Hangzhou, Zhejiang, 310058, P. R. China; 2 Department of Neurobiology, Key Laboratory of Medical Neurobiology of the Ministry of Health of China, Zhejiang University School of Medicine, Hangzhou, Zhejiang, 310058, P. R. China; 3 Department of Physiology, Yong Loo Lin School of Medicine, National University of Singapore, Singapore; 4 Department of Toxicology, Hangzhou Normal University School of Medicine, Hangzhou, Zhejiang, China

## Abstract

Hepatocellular carcinoma (HCC) is one of the most lethal and prevalent malignancies, worse still, there are very limited therapeutic measures with poor clinical outcomes. Dietary restriction (DR) has been known to inhibit spontaneous and induced tumors in several species, but the mechanisms are little known. In the current study, by using a diethylnitrosamine (DEN)-induced HCC mice model, we found that DR significantly reduced the hepatic tumor number and size, delayed tumor development, suppressed proliferation and promoted apoptosis. Further transcriptome sequencing of liver tissues from the DEN and the DEN accompanied with DR (DEN+DR) mice showed that DEN induced profound changes in the gene expression profile, especially in cancer-related pathways while DR treatment reversed most of the disturbed gene expression induced by DEN. Finally, transcription factor enrichment analysis uncovered the transcription factor specificity protein 1 (SP1) probably functioned as the main regulator of gene changes, orchestrating the protective effects of DR on DEN induced HCC. Taken together, by the first comprehensive transcriptome analysis, we elucidate that DR protects aginst DEN-induced HCC by restoring the disturbed gene expression profile, which holds the promise to provide effective molecular targets for cancer therapies.

Liver cancer is the sixth most prevalent and the second most lethal cancer in the world^1^. In 2012, it was estimated worldwide that about 782,000 new liver cancer cases occurred and nearly 746,000 people died of it^2^. Despite aggressive conventional therapies, the 5-year survival rate of individuals with liver cancer is only 8.9%^3^. Hepatocellular carcinoma (HCC) is a cancer of the parenchyma cells in liver and accounts for 83% of liver tumors^4^,^5^, which often occurs as a subsquent lesion from chronic liver diseases caused by a variety of risk factors including infection with viruses like hepatitis B and C, aflatoxin, obesity, alcohol consumption, tobacco usage and so on^6^,^7^. Since HCC is of such high risk for humam life, extensive studies have been conducted to understand the underlying molecular mechanisms for HCC, as well as finding potential therapeutic measures. The diethylnitrosamine (DEN)-induced HCC in mouse is one of the most frequently used animal models in liver cancer researches^8^,^9^, which has a gene expression profile similar to human HCC^10^. The formation of alkyl DNA adducts, which causes genome instability, and eventually results in the transformation of preneoplastic or neoplastic cells, has been considered as the key event in DEN-mediated tumorigenesis^11^,^12^,^13^.

Dietary restriction (DR) with a reduction about 10–40% intake of an ad libitum (AL) diet induces profound effects on animals at levels from the transcriptome to whole animal physiology and behavior^14^. Accumulative evidences indicate DR extends lifespan, delays aging and prevents age-related diseases in many species^15^,^16^,^17^. The broadly positive impacts of DR are mainly associated with dietary interventions including reduced daily food/calories intake, intermittent fasting, reduced protein or essential amino acid intake^18^,^19^.

In 1909, the effects of DR on tumors were investigated for the first time by Moreschi *et al*. who demonstrated that DR retarded the transplanted tumor growth in rats^20^. This finding triggered the century-long investigation studies regarding the role of DR in cancer prevention and treatment^21^. Numerous experiments in rodents have proved that DR reduces the incidence of tumor, delays tumorigenesis and improves survivals of animals with spontaneous and chemically induced tumors^22^,^23^,^24^. Mechanistic studies have prompted the role of DR in reducing carcinogen-DNA adducts, elimilating reactive oxygen species and maintaining genome stability as well as affecting physiological and biochemical processes including proliferation, inflammation, angiogenesis, autophagy and apoptosis^25^,^26^,^27^. In addition, several DR mimetics, such as resveratrol, metformine, and rapamycin, have been successfully used in combination with chemotherapies for clinical cancer therapies, signifying the potential application of DR in clinical settings^28^. However, the detailed molecular mechanisms of the positive effects of DR on primary tumor *in vivo* is still far from clear.

During the past decade, next-generation sequencing (NGS) has become an efficient and accurate method in molecular diagnostics by providing comprehensive genomic aberrations in tumors for both research and clinical purposes^29^,^30^. NGS encompasses a set of different techniques including whole genome/exome sequencing, RNA sequencing (RNA-Seq) and targeted sequencing of specific panels of genes^31^. By using the RNA-Seq platform, not only gene expression can be accurately and sensitively measured, aberrant fusions or chimeras, alternative splicing, aberrant gene editing, and RNA polyadenylation can also be determined^32^. Thus, major driver genes and associated oncogenic pathways operating in cancers could be discovered, which provided a comprehensive picture of the cancer transcriptome and pointed to the direction for further molecular mechanistic studies^33^,^34^.

In our study, by using the DEN-induced HCC model in C57BL/6 mice, we analyzed the global gene expression profiles of DEN and DEN+DR mice using the Illumina HiSeq-based RNA-Seq in an effort to identify new leads for the underlying molecular mechanisms for the protective effects of DR on cancer. As reported here, while DEN induced profound disturbances in gene expression of the mice model, DR could reverse most of the changes, thus conferring the protective effects on mice.

## Results

### Effects of DR and DEN on body weight, organ coefficient

**Figure 1:**
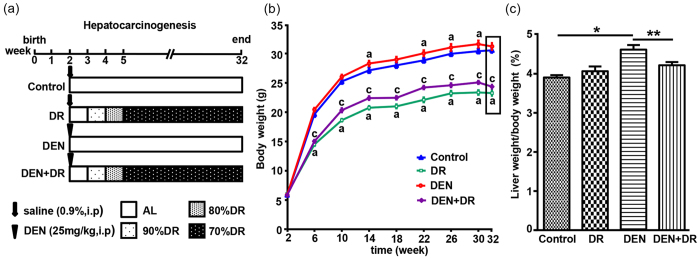
The effects of DEN, DR and DEN+DR on body weight and organ coefficient. (**a**) Flow chart for the experimental design. (**b**) Effects of DEN, DR and DEN+DR on body weight (n = 12 to 13), ^a^*P* < 0.05 compared with control, ^c^*P* < 0.05 compared with DEN. (**c**) Effects of DEN, DR and DEN+DR on organ coefficient in groups (n = 12 to 13), *P < 0.001, **P < 0.05.

To exposit the protective effects of DR on DEN-induced HCC, two-week-old male C57BL/6 mice were subjected to a multistep diet protocol with a final 70% DR or an AL diet for 30 weeks after 25 mg/kg DEN administration (Fig. 1(a)). Compared with AL feeding, restriction of food resulted in body weight decrease by approximate 20–30% during the experiment period. In contrast, while DEN caused obvious weight increase, all DEN-treated mice suffered a sudden decline in body weight at 32 weeks of age (Fig. 1(b)). Correspondingly, DEN group displayed much higher liver/body weight ratios compared with the control group (*P* < 0.05), which might be due to the DEN induced liver damage. Despite the reduced body weight in DR mice, no diffirence was found between control and DR mice for their liver/body weight ratios. On the other hand, the liver/body weight ratios of DEN+DR mice were decreased compared to DEN mice, indicating that DR probably alleviated the tumor burden (*P* < 0.05) (Fig. 1(c)).

### DR prevents DEN-induced hepatocarcinogenesis

**Figure 2:**
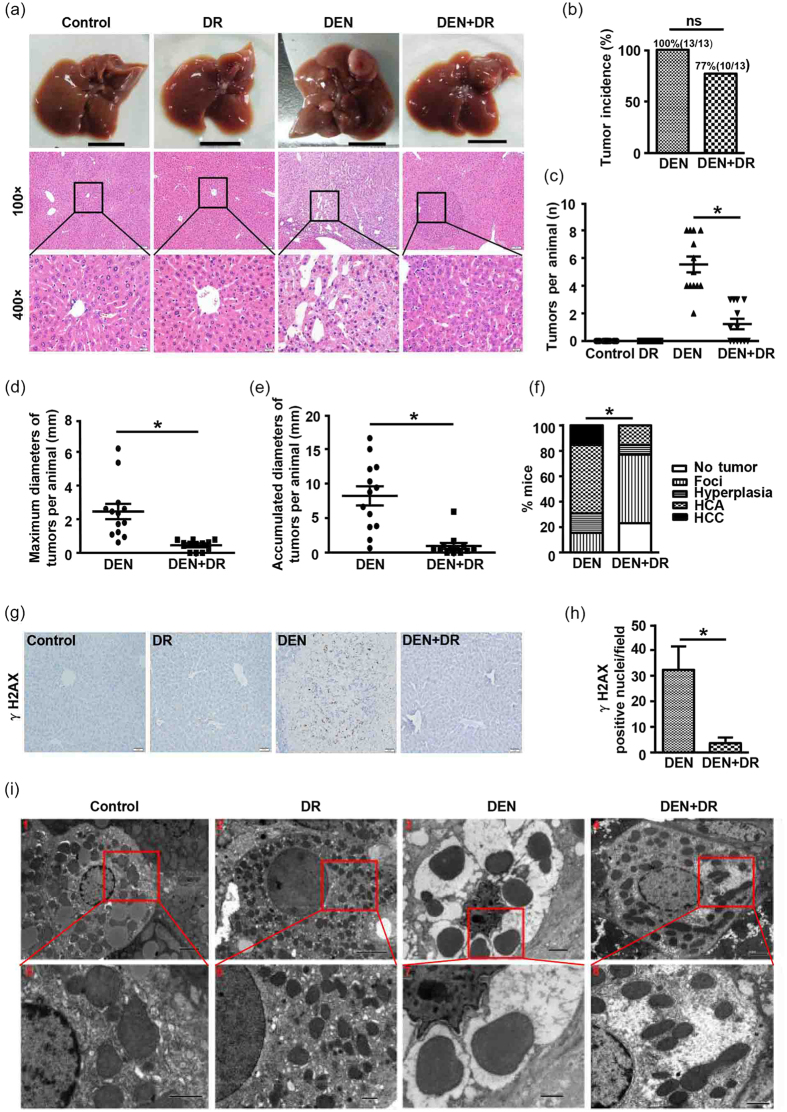
DR protects mice from DEN-induced HCC. (**a**) Representative macroscopic images and HE-stained liver sections of mice from different groups at 32 weeks of age [scale bar, 1 cm, 100 μm (100×), 20 μm (400×)]. (**b**) Tumor incidence in DEN and DEN+DR group, statistical analysis was conducted using the fisher exact test (n = 13). (**c**) Numbers of macroscopically detectable nodules on the liver surface of mice in each group (n = 12 to 13), *P < 0.001. (**d**,**e**) The maximal diameters and accumulated diameters of tumors per mouse were shown (n = 13), *P < 0.001. The diameters of nodules in each mouse were assessed by either vernier caliper for macorscopic nodules at liver surface or calibrated software for inner nodules by histological observation. (**f**) Percentage of mice with foci, hyperplasia, HCA and HCC, comparison between DEN and DEN+DR group was performed with the Mann-Whitney *U* tests (n = 13), *P < 0.01. Two 4-mm H&E-stained sections per liver were scored, the pathologic grade for each mouse was predicated by the most aggressive tumor in the fields. (**g**) Detection of hepatocytes with DNA damage by γH2AX immunostaining (Scale bar, 50 μm). (**h**) Quantification of γH2AX immunostaining, data from five 200× fields per liver were shown as the mean ± SEM (n = 3), *P < 0.05. (**i**) Representive electron microscopic images of the livers. Samples from DEN and DEN+DR groups were collected from macroscopic nodules at liver surface (n = 3), and the photographs were selected from more than ten coincident fields and shown at their indicated magnifications [(Scale bar, 2 μm (1, 3, 4), 5 μm (2), 1 μm (5, 6, 7, 8)].

At 32 weeks of age that is 30 weeks after DEN administration, all mice in the DEN group developed visible tumors while macroscopically detectable nodules were rarely found on liver surface of DEN+DR mice, and no nodules were detected in control or DR groups. As expected, histopathological characterization verified that HCC arose in DEN mice, but DEN+DR mice only displayed typical features of lower-grade foci (Fig. 2(a)). Combining the macroscopic observation and histopathological results, although no statistical significance for tumor incidence was found between DEN and DEN+DR group, three mice from DEN+DR group (3 out of 13) were classified as no tumor formation (Fig. 2(b)). Visible tumor nodules on the liver surface were counted for each mouse, and DEN mice developed remarkably more hepatic tumors than the DEN+DR ones (Fig. 2(c)). Sizes of the tumors were also measured, and it was clear that DEN mice beared strikingly larger tumors than DEN+DR mice (Fig. 2(d,e)). According to the international nomenclature for classification of rodent tumors^35^, liver histologic lesions were classified as foci, hyperplasia, hepatocellular adenoma (HCA), and more aggressive HCC. Based on such classification, by the end of 32 weeks of age, most DEN+DR mice developed foci (54%) whereas most DEN mice accelerated to HCA (54%) and more aggressive HCC (15%) (Fig. 2(f)). Since one of the major events for DEN-induced carcinogenesis is the formation of DNA adducts, and γH2AX has been recognized as a marker for DNA damage, we examined the liver tissues from mice for the evidence of DNA damage. As is shown in Fig. 2(g), livers of DEN mice contained many hepatocytes with γH2AX-positive nuclei indicating that DNA damage was occurring at one stage of tumorigenesis. And interestingly, much less γH2AX-positive nuclei in hepatocytes were seen in DEN+DR livers (Fig. 2(h)). Subcellular structures of hepatocytes were also observed by electron microscope assay (Fig. 2(i)). Hepatocytes from DR mice had similar cellular structure as control. In contrast, hepatocytes from DEN liver displayed necrotic morphological changes including disappeared cell boundary, broken membranes, muddy cytoplasm, inflated organelles and wrinkled nucleus. Surprisingly, subcellular structures of hepatocytes in DEN+DR liver were almost the same as those in control and DR groups.

### DR suppresses proliferation and promotes apoptosis

**Figure 3:**
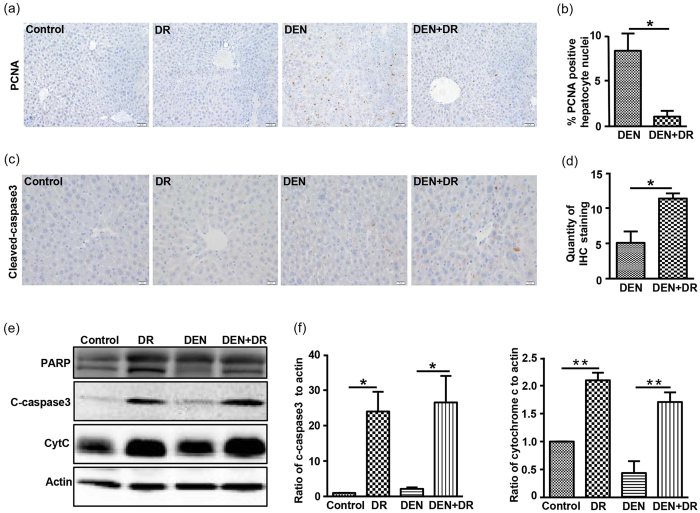
DR inhibits proliferation and induces apoptosis in hepatocytes. Representative images (**a**) and quantification (**b**) of proliferating hepatocytes by PCNA immunostaining of liver section, five 200× fields were counted for each liver section, and data were presented as the mean ± SEM (n = 3) (Scale bar, 50 μm), *P < 0.05. Representative images (**c**) and quantification (**d**) of cleaved-caspase3 immunostaining of liver sections, five 200× fields were counted for each liver section, and data were presented as the mean ± SEM (n = 3) (Scale bar, 20 μm), *P < 0.05. Representative immunoblot images (**e**) and quantification (**f**) of apoptosis-related proteins in liver lysates (n = 3), *P < 0.05, **P < 0.01.

Tumorigenesis is considered as a process of chronic damage including cycles of cell death and death-driven compensatory proliferation^36^,^37^. Proliferating cell nuclear antigen (PCNA) is a classical marker of liver damage and a sign of HCC^38^. In contrast to the normally quiescent hepatocytes in control mice, livers of DEN mice displayed a significant increase in proliferating hepatocytes as shown by positive staining of PCNA, and such proliferation was inhibited by DR as few PCNA-positive nuclei were observed in DEN+DR livers (Fig. 3(a,b)). On the other hand, DR increased apoptosis in livers suggested by the immunostaining of cleaved-caspase3 (Fig. 3(c,d)). Up-regulation of cytochrome C, cleaved-PARP and cleaved-caspase3, which are responsible for mitochondria dependent apoptosis, futher supported our argument that DR induced apoptosis in DEN mice (Fig. 3(e,f)).

### The effects of DEN on the transcriptomic signatures

**Figure 4:**
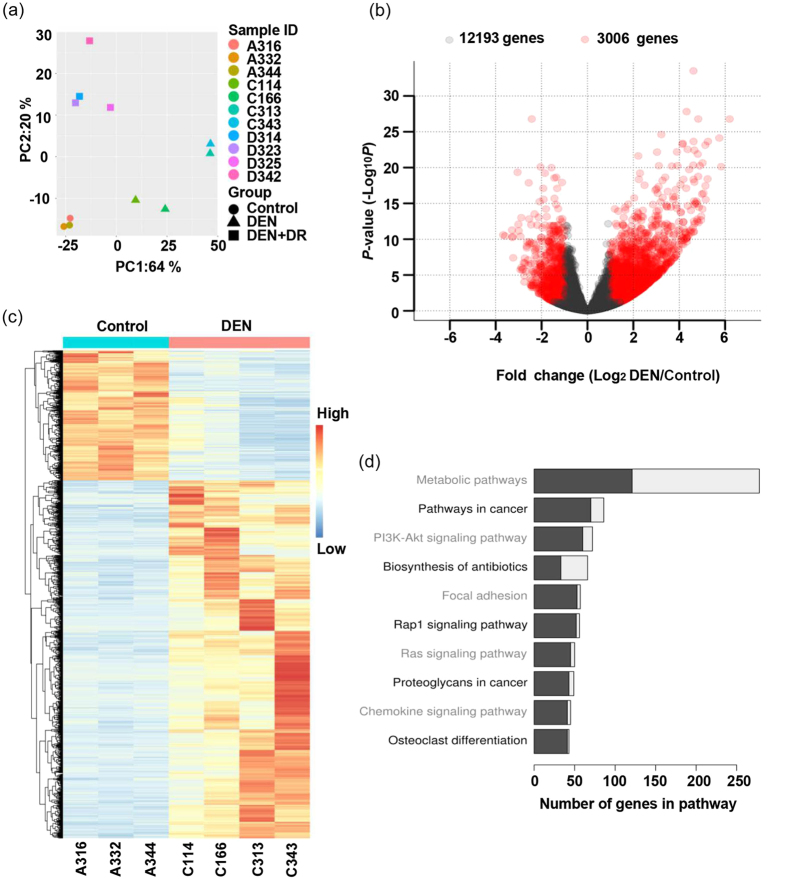
DEN induces profound changes in gene expression profile. (**a**) Principal component analysis of gene signatures of each mouse. (**b**) Volcano plot illustrated the fold change and statistical significance of genes in DEN group compared with the control group. (**c**) Heatmap showed the expression profiles of control and DEN mice. (**d**) Top ten pathways affected by DEN from KEGG enrichment analysis, black bars represented the genes up-regulated by DEN, grey bars represented the genes down-regulated by DEN compared with control.

Total of 11 samples (Control: 3; DEN: 4; and DR+DEN: 4) were subjected to RNA-seq. The obtained expression profiles were plotted by principal component analysis. Samples within one group shared much similarities, and groups distinguished well from each other despite variance still existed in DEN treated groups, which might be a result of complexity of carcinogenesis (Fig. 4(a)). Differential expression analysis revealed 3,006 genes were perturbed by DEN and 12,193 genes remained unchanged (Fig. 4(b)). Heatmap clearly demonstrated the significant difference of gene expressions between the DEN and control groups (Fig. 4(c)). KEGG pathway analysis showed that the most significantly affected pathways were metabolic and cancer pathways, as well as pathways that regulate cell adhesion, growth, communication and differentiation (Fig. 4(d)). Except for metabolic and biosynthesis antibiotic pathways, most differential expression genes in other eight pathways were up-regulated by DEN.

### Perturbation of gene expression are normalized by DR

**Figure 5:**
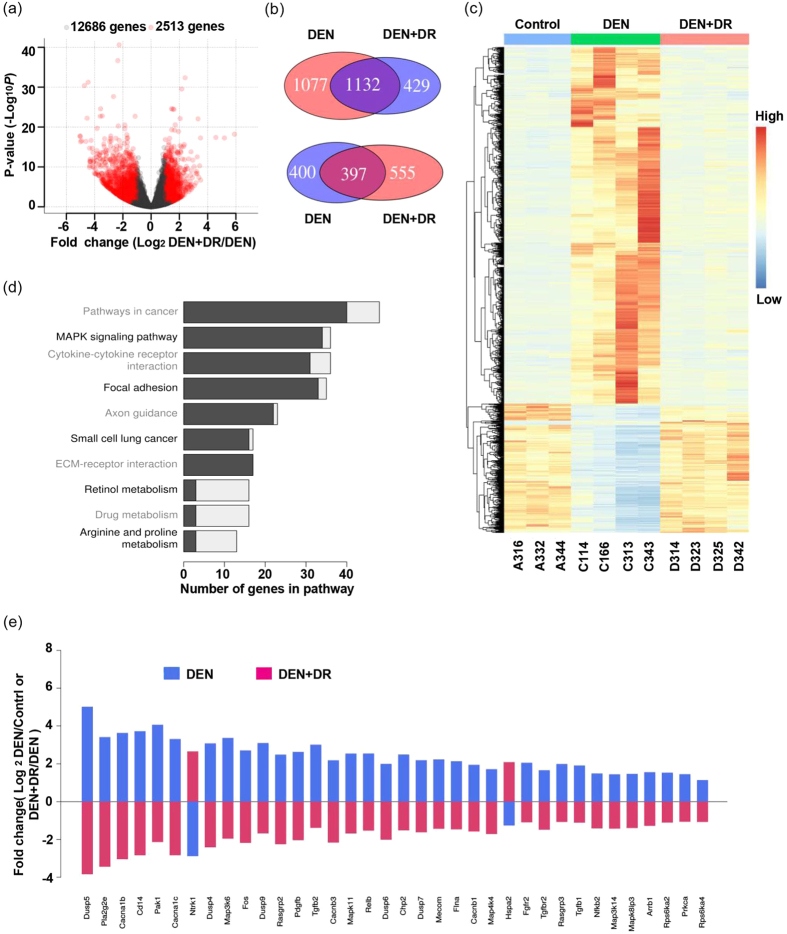
DR reversed the changed gene expression induced by DEN. (**a**) Volcano plot illustrated the fold change and statistical significance of genes in DEN+DR group compared with the DEN group. (**b**) Venn diagram showing the overlap of genes regulated by DEN and DEN+DR to opposite direction. The pink rounds represented the up-regulated genes and blue rounds represented down-regulated genes. (**c**) Heatmap showed the expression of overlapping genes between DEN and DEN+DR group. (**d**) KEGG enrichment of genes regulated by DEN and DEN+DR in opposite direction. Black bars represented the genes up-regulated in DEN and down-regulated in DEN+DR, grey bars represented the genes down-regulated in DEN and up-regulated in DEN+DR. (**e**) Comparison of gene expression in MAPK pathway between DEN and DEN+DR: most of the genes were up-regulated by DEN and reversed by DR, and a few genes were regulated in the contrary way.

Comparation of the RNA-seq results from DEN+DR and DEN mice showed 2,513 genes were significantly changed while 12,686 were not (Fig. 5(a)). Interestingly, these DEN+DR signatures significantly overlapped with DEN signatures, as showed in venn diagram, 1,132 genes up-regulated by DEN were down-regulated by DR, and 397 genes down-regulated by DEN were up-regulated by DR (Fig. 5(b)). This phenomenon can be more clearly visualized in the heatmap, which indicated DR reversed the expression of many gene signatures disturbed by DEN (Fig. 5(c)). KEGG enrichment analysis was performed to identify the mostly affected pathways, and those pathways related to cancer, cell metabolism, growth and communication were also identified (Fig. 5(d)). One of the pathways, e.g., MAPK pathway, was subjected to further analysis (Fig. 5(e)), as was shown, DR reversed the overexpression of genes perturbed by DEN such as *fos, mecom, dusp5, dusp6, dusp7, dusp9* and so on.

### Gene network modeling of transcriptional signatures in DEN and DEN+DR mice

**Figure 6:**
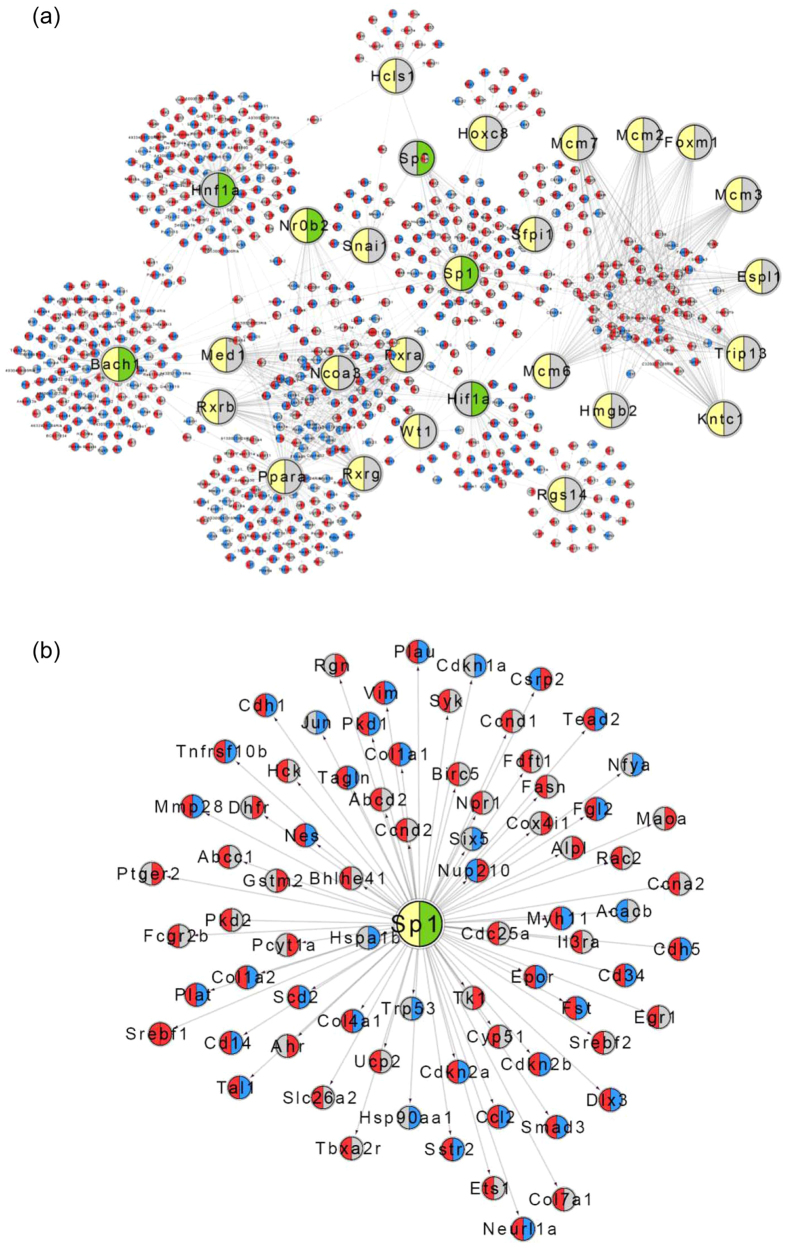
Gene networks and the key-driver transcription factor (TF) in DEN and DEN+DR. (**a**) TFs and gene subnetwork constructed for DEN and DEN+DR mice. (**b**) The genes enriched in SP1 networks. Larger nodes represents TFs; grey nodes are network genes that are not affected by DEN or DEN+DR; left and right halves of each node, if colored, denotes genes affected by DEN (left) and DEN+DR (right), respectively; red and blue colors denotes increased and decreased expression, respectively. Direction of change for the DEN group is determined by comparison with the control group; direction of change for the DEN+DR group is determined by comparison with the DEN group.

To identify the key regulators of genetic signatures in DEN and DEN+DR mice, we employed the RegNetwork with an integrated database of transcriptional and post-transcriptional regulations. Using these networks and the transcription factor enrichment analysis, most of the signature genes were enriched in 24 transcription factor (TF) sets, and three of them highly overlapped with the signature genes of both DEN and DEN+DR groups (Fig. 6(a)). Among the three TFs, SP1 has been reported to play an important role in HCC^39^, as is shown, genes including *cdkn2b, smad3, cdkn2a, col4a1*, which were enriched in cancer and MAPK pathways, were down-stream genes of SP1 (Fig. 6(b)).

## Discussion

The protective effects of DR on carcinogenesis have long been recognized, although the detailed underlying molecular mechanisms remain to be fully elucidated. In this study, we evaluated the occurrence and development of liver tumors under DR administration, which is the first comprehensive overview of the DR protective effects on tumorgenesis including its number, size, pathological grade, and the damage of its subcellular structures. By sequencing of transcriptome, we found that DEN induced profound changes of genes involved in metabolism, cancer, cell growth, differentiation and communication. Not surprisingly, it was found that DR reversed the genes aberrantly regulated by DEN. To be noted, genes up-regulated by DEN in the ‘MAPK pathway’, which was reported to be of great importance in HCC, were completely restored by DR. Using gene network analysis, we also identified transcription factor SP1 as the potential major orchestrator in DEN and DR-induced events.

The transformation of preneoplastic or neoplastic cells from normal hepatocytes caused by genetic alteration is considered as the initial step of the multistage carcinogenesis, which was irreversible in DEN induced liver cancer^40^. It has been reported that such initiated hepatocytes, with the putative marker glutathione S-transferase Pi (GSTP) could be found within two weeks after DEN administration^41^. Those initiated hepatocytes then would undergo frequent proliferation to expand their numbers and thus more and more DNA damage was accumulated. This would result in foci formation and persistent nodules, which would eventually lead to HCC^42^. In our study, aggressive tumors with increased DNA damage and hepatocyte proliferation were found in DEN mice while DEN+DR mice had reduced tumor number as well as less DNA damage in cells (Figs 2 and 3). Such observation was in consistent with previous reports in rodents showing that DR inhibited proliferation and induced apoptotic cell death to selectively eliminate senescent, preneoplastic, or superfluous cells to maintain cell homeostasis^43^.

Regardless of the etiology, the majority of HCC cases occur against a background of chronic inflammation^44^,^45^,^46^, which impacts every single step of the tumorigenesis process ranging from initiation to progression^47^. In our experiment, alleviated inflammation such as macrophage infiltration in DEN+DR group compared with DEN group was observed, as detected by F4/80 staining (Fig. S1(a)). Moreover, as indicated by the nuclear accumulation of NF-κB2 and NF-κB (p65) (Fig. S1(b)), a large number of cells within the tumors of DEN mice displayed the activation of NF-kB signaling pathway; in contrast, only a few cells within the tumors of DEN+DR mice exhibited the activation of NF-κB. These results all suggested that DR alleviated the inflammation and suppressed the NF-κB signaling in DEN induced HCC model. It is hypothesized that DR could regulate cell proliferation and apoptosis by targeting the NF-κB signaling pathway, which is regarded as one of the important pathways linking inflammation and cancer by affecting process including cell proliferation, apoptosis, cellular senescence and angiogenesis^48^,^49^,^50^. We believed that inflammation should be an important direction for further research in the protective role of DR in cancer.

In our study, by RNA-Seq analysis, we revealed DEN disturbed the expression of a large number of genes, among which nearly 75% were up-regulated by DEN. Functional analysis showed that most of these genes were involved in pathways including ‘metabolic pathway’, ‘pathways in cancer’, ‘ras pathway’, ‘PI3K-AKT pathway’, ‘rap1 pathway’ and ‘focal adhesion’. As expected, many of the pathways are known major players in cancer. For example, the ras signaling, which regulates cell growth, apoptosis, and differentiation by activating the RAF-MEK-ERK/MAPK cascade, has been proved to be frequently activated by mutation of ras family and causally linked to DEN induced HCC^51^.

More importantly, our study revealed the broad impacts of DR on fundamental aspects of the gene expression disturbed by DEN. Mice from DEN and DEN+DR groups shared an overlap of 1,529 genes, which prominently interrelated cancer-related signalings including ‘pathways in cancer’, ‘MAPK signaling pathway’, ‘focal adhesion’, ‘small cell lung cancer’, ‘ECM receptor interaction’ and hepatic function-related pathways including ‘retinol metabolism’, ‘drug metabolism’, and ‘arginine and proline metabolism’. Among genes enriched in those pathways, some have been confirmed to be closely associated with HCC. For examples, *fos and mecom*, which are oncogenes involved in proliferation and differentiation, have been reported to promote HCC when overexpressed^52^,^53^; *pparg*, which was down-regulated in DEN and up-regulated in DEN+DR (data not shown), is a gene responsible for coding protein PPARγ that has been proved to protect aginst hepatocellular carcinoma by inhibiting cell growth, migration, invasion, metastasis and inducing apoptosis both *in vitro* and *in vivo* studies^54^,^55^,^56^. Furthmore, Yu *et al*. showed *PPARγ* preventd DEN-induced HCC using a *PPARγ*-deficiency mouse model, which served as a solid evidence for our results and suggested the significant role of DR in inhibiting HCC^57^. Specifically, we found DR restored many genes in the ‘MAPK signaling’ including *fos, mecom, map3k6, mapk11, map4k4, rps6ka2, rps6ka4, cacna1b, cacna1c, cacnb3, cacnb1, dusp5, dusp9, dusp4, dusp7 and dusp6*, which was consistent with previous studies showing DR supressed the MAPK signaling^58^,^59^. As an important signaling in tumor development, MAPK signaling promotes cancers including DEN induced HCC by directing cellular responses including proliferation, survival, differentiation and migration^60^,^61^,^62^,^63^,^64^. Moreover, the RAS-RAF-MEK-ERK/MAPK cascade is one of the main targets of Sorafenib which is the only systematic therapy currently effective for advanced HCC. Taken together, we proposed that the effects of DR on genes enriched in ‘MAPK signaling’ in DEN model played an important role in its protective role in HCC and underlied the potential value of DR in clinical therapies.

By transcription factor enrichments analysis, many cancer-related genes from the overlap of DEN and DEN+DR signature genes were enriched in SP1 network. For examples, both *plau* and *vim* are reported to be invloved in HCC development^65^,^66^. In previous studies, it has been shown that SP1 was abnormally activated in tumor tissues^67^ and affected tumor progression and its clinical survival by regulating tumor cell proliferation, invasion, angiogenesis, and other biological functions^68^,^69^,^70^. As one of the main targets for MAPKs, the activation of SP1 could be modulated by both ERK and JNK in MAPK signaling. For instance, MAPK upregulated expression of *plau* through SP1 phosphorylation^71^. Thus, we propose that SP1 is a major player of effects of DR on transcriptome in DEN induced HCC, which was probably associated with its role in the MAPK signaling.

In summary, we confirmed that DR inhibited the DEN induced hepatocarcinogenesis and proposed that the aberrant regulation of proliferation and apoptosis was causally linked with this inhibitory role. By RNA-Seq, we revealed that DEN reprogrammed the transcriptome to promote the tumor growth. Remarkably, our study demonstrated that DR reversed the genetic changes perturbed by DEN and thus prevented the DEN induced tumor development. In addition, our findings indicated that DR induced transcriptomic remodeling in DEN induced HCC by engaging transcription factors such as SP1. Therefore, our experiments uncovered the clues to identify the mechanisms and provided molecullar supports for DR in cancer treatment.

## Methods

### Animals and treatments

C57BL/6 mice were bred by Zhejiang Chinese Medical University Laboratory Animal Center and maintained in a specific pathogen-free environment. Animals were housed in individual ventilated cages (no more than five animals per cage) under standard laboratory conditions (temperature 24 ± 0.5 °C, relative humidity 55 ± 5%, and a 12-h dark/light cycle). Male mice were injected intraperitoneally with 25 mg/kg body weight of DEN (Sigma-Aldrich, Cat# N0756) at the age of 2 weeks and allowed to grow until 32 weeks of age. Littermate controls were injected with equal volume of saline. DR was carried out from age of 3 weeks in a multistep-advanced process: 90% of AL-fed for one week followed by 80% for one week and finally stabilized at 70% till the ending point. The amount of food taken by DR mice per day was calculated based on the food consumed by corresponding control mice the day before. Food was given between 5:00 to 6:00 pm every day. Body weight of each mouse was recorded once a week. All animals had free access to water. All experiments were performed under the Guide for the Care and Use of Laboratory Animals (The National Academy Press, 2011) and approved by both Laboratory Animal Committee of Zhejiang University and Zhejiang Chinese Medical University.

### Sample preparation

Mice were killed for examination of liver tumors after overnight starvation. Unbroken livers were removed, weighed and photographed with measuring scale. Sections from left lateral lobe were fixed in 10% formalin for hematoxylin-eosin (HE) staining or immunohistochemistry (IHC), a little piece of tissue was fixed in 2% glutaraldehyde solution for transmission electron microscope (TEM), and the remaining tissue was snap frozen in liquid nitrogen and then stored at −80 °C until assayed.

### Analysis of liver tumor

All visible nodules at liver surface were counted for each mouse. Tumors of each mouse were measured by vernier caliper or calibrated software according to HE staining. Liver histologic lesions were classified according to standardized and internationally accepted nomenclature for classification of rodent tumors^35^ by two different experienced pathologists in a blind fashion.

### Immunohistochemistry

Immunohistological staining was performed on paraffin embedded sections following standard protocols. Briefly, after deparaffinization of specimens, the antigen was retrieved by incubation in boiling citric acid buffer (pH = 6.0) for 2 min, subsequently, endogenous peroxidase was quenched with 3% hydrogen peroxide at room temperature for 15 min. After being blocked, the sections were incubated with primary antibody-recognizing PCNA (Abcam, Cat#2426) at 1:1000 dilution, F4/80 (Abcam, Cat#15694) at 1:50 dilution, γH2AX (Cell Signaling Technology, Cat#9718) at 1:1000 dilution, Cleaved-caspase3 (Cell Signaling Technology, Cat#9661) at 1:100 dilution, NF-κB2 (Cell Signaling Technology, Cat#3017) at 1:500 dilution, NF-κB (p65) (HuaAn Biotechnology, China, Cat#ER0815) at 1:100 dilution overnight at 4 °C and then with a MaxVision+Syetem HRP-Polymer (Fuzhoumaixin, China, Cat#5001) at room temperature for 30 min. At last, sections were incubated with a Liquid DAB Substrate Chromogen System (Fuzhoumaixin, China, Cat#0015). Immunohistochemistry staining was photoed by a Olympus microscope (Olympus, Japan) and quantified with Image Pro Plus 6 software.

### Immunoblotting

Liver tissues, stored at −80 °C, were homogenized in ice-cold RIPA buffer (Beyotime Institute of Biotechnology, Cat#P0013B) with 1× protease inhibitor cocktail (Sigma, Cat#P8340) (pH = 7.4) for 25 min, and the remaining debris was cleared by subsequent 10 min centrifugation at 14,000 *g*. The protein concentrations were determined using a BCA protein assay kit (Beyotime Institute of Biotechnology, Cat#P0010). About 30 μg of total protein per lane was diluted in standard SDS sample buffer and subjected to electrophoresis on 10% SDS-polyacrylamide gels, proteins were then transferred to polyvinylidene difluoride membranes (Millipore, Cat#IPVH00010), blocked with 5% BSA (Sangon Biotech, Cat#9048-46-8) in Tris-buffered saline containing 0.1% Tween-20 (TBST) for 2 h at room temperature and incubated with the primary antibody (PARP, Cell Signaling Technology, Cat#9542, 1:1000 dilution; Cleaved-caspase 3, Cell Signaling Technology, Cat# 9661, 1:500 dilution; Cytochrome C, Abcam, Cat#133504, 1:1000 dilution; Actin, Abcam, Cat#3280, 1:500 dilution) over night at 4 °C. The membranes were then washed with TBST for three times and incubated with IRDye^®^ 680LT Goat anti-Mouse IgG (Li-Cor Biotechnology, Cat#926-68020, 1:10000 dilution) or RDye^®^ 800CW Goat anti-Rabbit IgG (Li-Cor Biotechnology, Cat#926-32211, 1:5000 dilution) for 2 h at room temperature. Protein bands were visualized using an Odyssey Infrared Imaging System (Li-Cor Biosciences). Quantity One sofware (BioRad) was used for densitometric scanning.

### Transmission electron microscopy

Liver tissues cut into pieces of approximately 1 mm cubes were fixed with 2.5% glutaraldehyde at 4 °C for at least 2 h. After three times washing in 0.1 M PBS, samples were post-fixed in 1% osmium tetroxide for 2 h at room temperature, washed in 0.1 M PBS (15 min × 2), incubated in 2% uranyl acetate for 30 min and then dehydrated in increasing concentrations of ethanol (15 min each, 50, 70, and 90%; 10 min × 2, 100%). After dehydration, samples were put into 100% acetone for 20 min at room temperature, infiltrated in a 1:1 mixture of 100% acetone and 100% Lowicryl K4M resin for 2 h at room temperature, followed by embedding in 100% Lowicryl K4M resin and polymerization (37 °C, 24 h; 45 °C, 24 h; 60 °C, 48 h). An ultramicrotome with a glass knife was used to take ultrathin sections. At last, sections were stained with 4% uranyl acetate for 20 min and lead citrate for 5 min. Sections were examined with Electron Microscope (JOEL, Japan) operating at 80 kV, with images recorded with a coupled camera.

### RNA isolation and Sequencing

Total RNA was isolated from 20 mg liver tissues using RNeasy Mini Kit (QIAGEN, Cat#74104) following the manufacturer’s protocol. Quantity of RNA was checked by Nanodrop (Thermo Fisher Scientific, MA, USA), Qubit 2.0 (Thermo Fisher Scientific, MA, USA) and an Agilent Bioanalyzer 2100 (Agilent Technologies, CA, USA). The RNA-Seq libraries were prepared following the standard Illumina protocol (http://support.illumina.com/sequencing/documentation.ilmn). Briefly, mRNA was enriched from 1 μg total RNA by polyA selection using oligo (dT) beads, then fragmented by fragmentation buffer, and reverse transcribed using random hexamer-primers to generate first-strand cDNA. Second-strand cDNA was then generated using RNase H and DNA polymerase. Next, cDNA was subjected to purification by AMPure XP beads, end repair, A-base addition and ligation of the Illumina-indexed adaptors. After enrichment through PCR, paired-end libraries were sequenced on the Illumina HiSeq 4000 platform (2 × 150 bp read length).

### Sequencing data analysis

After filtering out the adaptor sequences, low quality reads and duplicate reads, the clean reads were used for alignment. According to the workflow described by Love *et al*.^72^, reads were mapped to the mouse reference genome (GRCm38) using STAR-2.5.2a^73^. STAR was run in default parameters, and mouse genome annotation (GENCODE Version M9) was used as the junction annotation in index building step to improve matching accuracy^74^. Reads counting was conducted by ‘Genomic Alignments’^75^ with a mode from ‘Intersection-strict’. DESeq2 was used to normalize and compare read counts between DEN group and control group. The Benjamini-Hochberg method was used to correct multiple hypothesis testing by calculating false discover rate (FDR). Genes showing differential expression with FDR <0.05 and a fold change (FC) >2 were defined as a gene ‘signature’ for further testing for biological pathways and network analysis. The same analysis was applied to compare read counts between DEN+DR and DEN for identifying DR ‘signature’ genes.

### KEGG pathway analysis of signature genes

KEGG pathway enrichment analysis was performed by DAVID Bioinformatics Resources 6.8 as described (http://david.abcc.ncifcrf.gov/). EASE score was calculated for the enrichment *P*-values for each pathway. Results were corrected for multiple comparisons using the Benjamini-Hochberg method, with *P*-values <0.05 considered significant.

### Transcriptional enrichment analysis

DEN and DEN+DR signature genes identified from RNA-Seq analysis were classifed for their transcription factors (TF) using the RegNetwork, which integrates the knowledge-based regulatory relationships^76^. TF sets with down-stream gene numbers > 15 were selected for subsequent analysis. Fisher’s exact test was performed to calculate the enrichment *P*-values for each TF regulating gene set within each gene signature. Results were corrected for multiple comparisons using the Benjamini-Hochberg method. The TFs which regulated gene sets with a FDR < 0.1 and OR (odds ratio) > 1.5 were considered statistically significant. Such gene sets were chosen to construct the gene network, which was visualized with Cytoscape2.8.1^77^. MultiColorNodes plugin was used to display multiple colors in a single node^78^.

### Statistical analysis

Data were presented as mean ± SEM. After a normality of the distribution and the homogeneity of variance test of the data with the Levene test, comparisons between groups were performed with Student’s *t* test (two-tailed), except where indicated otherwise. *P*-values < 0.05 were considered statistically significant.

## Additional Information

**How to cite this article**: Duan, T. *et al*. Dietary restriction protects against diethylnitrosamine-induced hepatocellular tumorigenesis by restoring the disturbed gene expression profile. *Sci. Rep.* **7**, 43745; doi: 10.1038/srep43745 (2017).

**Publisher's note:** Springer Nature remains neutral with regard to jurisdictional claims in published maps and institutional affiliations.

## Supplementary Material

Supplementary Dataset 1
