# Supplementary material for: Dietary restriction protects against diethylnitrosamine-induced hepatocellular tumorigenesis by restoring the disturbed gene expression profile
Source: Sci Rep. 2017 Mar 6;7:43745. doi: 10.1038/srep43745 (PMC5338348; doi:10.1038/srep43745)
Supplement: Supplementary Dataset 1 [file srep43745-s1.doc]

**Dietary restriction protects against diethylnitrosamine-induced hepatocellular tumorigenesis by restoring the disturbed gene expression profile**

Ting Duan^1*^, Wenjie Sun^1*^, Mohan Zhang^1^, Juan Ge^1^, Yansu He^1^, Jun Zhang^1^, Yifan Zheng^1^, Wei Yang^2^, Han-ming Shen^3^, Jun Yang^4#^, Xinqiang Zhu^1#^, Peilin Yu^1#^


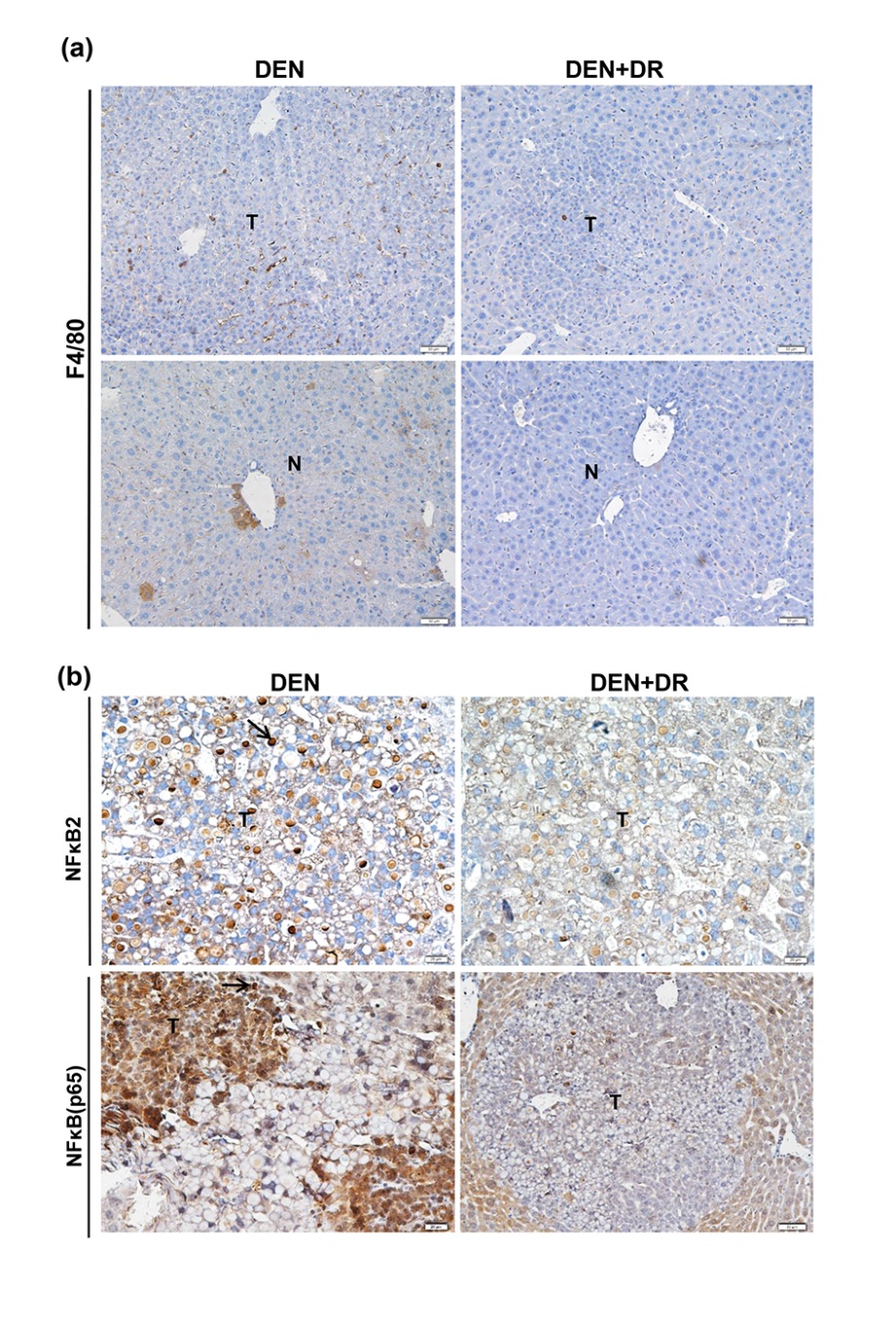


**Supplementary Figure 1.** Assessment of inflammatory state in liver of DEN and DEN+DR mice. (a) Representative immunostaining images of liver sections from DEN and DEN+DR mice for macrophage marker F4/80. (Scale bar, 50 µm). (b) Representative immunostaining images of liver sections from DEN and DEN+DR mice for NF-κB2 and NF-κB(p65), arrows indicate cells with nuclear accumulation of NF-κB2 and NF-κB(p65). (Scale bar, 20 µm). N (non-tumor region); T (tumor region).
